# Analysis of Airborne Particulate Matter (PM_2.5_) over Hong Kong Using Remote Sensing and GIS

**DOI:** 10.3390/s120606825

**Published:** 2012-05-25

**Authors:** Wenzhong Shi, Man Sing Wong, Jingzhi Wang, Yuanling Zhao

**Affiliations:** Joint Laboratory on Geo-Spatial Information Science, The Hong Kong Polytechnic University and Wuhan University, Hong Kong and Wuhan, China; E-Mails: lswzshi@polyu.edu.hk (W.S.); luckie-wang@hotmail.com (J.W.); zyl@whu.edu.cn (Y.Z.)

**Keywords:** aerosol optical thickness, GIS, particulate matter, remote sensing, visualization

## Abstract

Airborne fine particulates (PM_2.5_; particulate matter with diameter less than 2.5 μm) are receiving increasing attention for their potential toxicities and roles in visibility and health. In this study, we interpreted the behavior of PM_2.5_ and its correlation with meteorological parameters in Hong Kong, during 2007–2008. Significant diurnal variations of PM_2.5_ concentrations were observed and showed a distinctive bimodal pattern with two marked peaks during the morning and evening rush hour times, due to dense traffic. The study observed higher PM_2.5_ concentrations in winter when the northerly and northeasterly winds bring pollutants from the Chinese mainland, whereas southerly monsoon winds from the sea bring fresh air to the city in summer. In addition, higher concentrations of PM_2.5_ were observed in rush hours on weekdays compared to weekends, suggesting the influence of anthropogenic activities on fine particulate levels, e.g., traffic-related local PM_2.5_ emissions. To understand the spatial pattern of PM_2.5_ concentrations in the context of the built-up environment of Hong Kong, we utilized MODerate Resolution Imaging Spectroradiometer (MODIS) Aerosol Optical Thickness (AOT) 500 m data and visibility data to derive aerosol extinction profile, then converted to aerosol and PM_2.5_ vertical profiles. A Geographic Information Systems (GIS) prototype was developed to integrate atmospheric PM_2.5_ vertical profiles with 3D GIS data. An example of the query function in GIS prototype is given. The resulting 3D database of PM_2.5_ concentrations provides crucial information to air quality regulators and decision makers to comply with air quality standards and in devising control strategies.

## 1. Introduction

Airborne Particulate Matter (PM) refers to particles suspended in the air in either liquid or solid form, which are highly heterogeneous in both time and space and are often observable as dust, smoke and haze. PM_2.5_ and PM_10_ are defined as particles with diameters of 2.5 μm or less, and 10 μm or less respectively, they are the standard concentrations used in the United States Environmental Protection Agency (EPA). Aerosol is defined as the total particles suspended in air with typical particle radius ranged from 0.05 to 15 μm [1]. Around 10% of the aerosols is produced by or is a result of human activities such as vehicular exhaust, burning of fossil fuel, construction, while the remaining 90% is produced by natural sources such as volcanic eruptions, sea spray and dust [2,3]. The scattering and absorption of light by the aerosol particles results in a degradation of visibility [4]. Satellite aerosol remote sensing provides Aerosol Optical Thickness (AOT) data, as a quantitative measurement of PM loadings in the atmosphere column [5]. To some extent, the AOT can be seen as an important indicator of air pollution and is the most readily recognized indication of the presence of particulate air pollution.

Airborne particulates can be inhaled by the human lungs, where they are absorbed into blood, and consequently are responsible for harmful health effects. The significance of adverse effects on our health depends on the size and composition of particulates. For instance, particles less than 2.5 μm (PM_2.5_) can penetrate deeper into the air sacs of human lungs and therefore pose the greatest harm to human health [6]. Environmental epidemiological studies have found particulate matters affect pulmonary function and can thereby induce respiratory diseases and adverse effects on public health and even premature death [7–9].

Elevated levels of PM_2.5_ over urban areas are often associated with both local sources of emissions and regional transport [10]. Although diesel vehicles are the main local sources of urban PM_2.5_ loads [11], regional transport and secondary transformation also account for a significant portion of PM_2.5_ levels. Numerous studies have been conducted to link the behavior of PM_2.5_ to meteorological data e.g., wind speed, wind direction, temperature, humidity, mixing height, precipitation, pressure and cloud cover [12,13]. Jung *et al.* [14] studied the atmospheric transport of PM_2.5_ in Ohio, United States, and found high concentrations of PM_2.5_ were particularly detected when the wind speeds were lower than 8 mph and the temperature was higher than 70 °F. Hien *et al.* [15] revealed that the fine particles were governed mainly by wind speed and temperature. Chiang *et al.* [16] found wind direction and relative humidity are highly correlated to fine particulates in winter.

Due to the temporal and spatial dependence of the pollutant, the characteristics of PM_2.5_ resolved in one region cannot be replicated to another region. Although there are some existing PM_2.5_ studies in Hong Kong [17–19], they are mainly focused on the chemical composition and only a few studies link pollutant characteristics to the meteorological parameters such as wind effects [20]. The extensive and comprehensive meteorology contribution to PM_2.5_ loadings is poorly understood in Hong Kong. Since understanding the pattern of pollutant and quantifying the relative contribution of different meteorological parameters are critical in developing control and mitigation strategies to safeguard public health, a detailed analysis of the temporal pattern of PM_2.5_ and the related meteorological contribution is imperative in Hong Kong. Thus, the objective of this study is to assess temporal and spatial patterns of PM_2.5_ in Hong Kong. The temporal variations of PM_2.5_ over urban areas in Hong Kong will be analyzed using ground-based data (meteorological and PM_2.5_ data), while the spatial patterns of PM_2.5_ will be derived from remote sensing and GIS approaches.

## 2. Data Collection

### 2.1. PM2.5 and Meteorological Measurements

**Figure 1.**
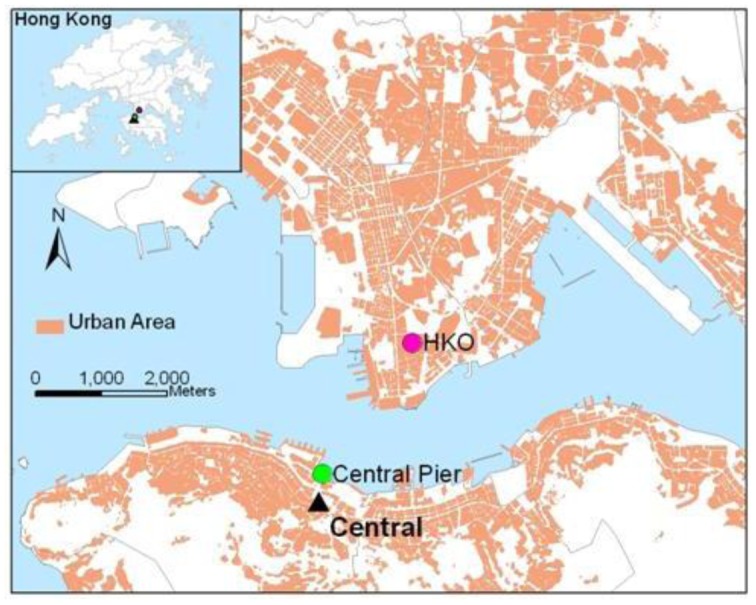
The locations of PM_2.5_ Central station, Central Pier and Hong Kong Observatory.

To characterize and analyze the PM_2.5_ concentrations in Hong Kong, the PM_2.5_ concentrations and meteorological data were acquired from the Hong Kong Environment Protection Department (HKEPD) and the Hong Kong Observatory (HKO) respectively. In this study, PM_2.5_ data recorded by Central station (22°16′54″, 114°09′29″) equipped with a TEOM Series 1400a monitor [21] are selected to represent PM_2.5_ concentrations over urban areas in Hong Kong. These data are represented for the pollution in Central Business District and are considered to have higher values than suburban and rural areas. Temperature, relative humidity, pressure, and precipitation were collected from the HKO (22°18′07″, 114°10′27″), which were used to represent the meteorological conditions for Central station (Figure 1). The wind speed and wind direction were collected from Central Pier monitoring station (22°17′20″, 114°09′21″) for representing the wind conditions for Central station as geographical proximity. These data are co-located in both space and time, which serve as the basis for statistical analysis.

### 2.2. MODIS AOT 500 m Image

The MODerate Resolution Imaging Spectroradiometer (MODIS) is a sensor aboard the TERRA and AQUA Earth observation system satellites. It is a multispectral (36 spectral wavebands span over the visible light, near infrared and infrared portion of the spectrum), multi-resolution (1 km, 500 m, 250 m) sensor dedicated to the observation of the Earth. However the coarse spatial resolution (10 × 10 km) of MODIS Aerosol Optical Thickness (AOT), namely MOD04 aerosol product [22] cannot provide detailed spatial variation for local/urban scale aerosol monitoring and is inaccurate over bright urban surfaces [23], Wong *et al.* [23,24] developed a modified Minimum Reflectance Technique (MRT) to derive AOT over both bright and dark surfaces (e.g., urban and vegetated areas) at the relatively high resolution of 500 m, for Hong Kong and the Pearl River Delta regions.

## 3. Methodology

### 3.1. Analyzing PM_2.5_ with Meteorological Data

In order to understand the interrelationship between PM_2.5_ and meteorological parameters, the correlations between them were first calculated. The diurnal patterns of PM_2.5_ concentration and meteorological data were also studied to understand their influences during summer and winter time. In addition, seasonal variations of PM_2.5_ as well as meteorological parameters were studied. The daily concentrations (24 hour average) of PM_2.5_ and meteorological parameters of 2007 and 2008 were calculated from the hourly data and then grouped into each season such as spring (March–May), summer (June–August), autumn (September–November) and winter (December–February).

### 3.2. Modeling PM_2.5_ Data with AOT Data

In contrast to ground level PM_2.5_ measurement, satellite remote sensing provides aerosol optical thickness to study urban air pollution with broad spatial coverage [25]. AOT is found to be dominated by near-surface emission except for long range dust events [26]. Recent studies have established quantitative relationships between MODIS derived AOT and PM_2.5_ using linear regression models. Wang and Christopher [27] achieved a correlation coefficient of 0.7 between satellite-derived AOT at 550 nm and PM_2.5_ measured at seven locations in Alabama, United States. Wong *et al.* [28] showed a good correlation between MODIS derived 500 m AOT and PM_2.5_ (r^2^ = 0.67), which demonstrated great potential for MODIS derived 500 m AOT as a good surrogate for PM_2.5_ monitoring. In this study, we attempted to model the 2D (image) and vertical distributions of PM_2.5_ which has not been done in any other study. The resulting 3D database of PM_2.5_ concentrations can be used for daily air quality monitoring in environmental authority. First, the aerosol extinction profile (σ_a_(z)) was modeled and the columnar AOT was divided into AOT_Δz_ at different elevations [29,30]. Then by utilizing the equation (PM_2.5_ = 63.66 × AOT + 26.56) developed by Wong *et al.* [28], the PM_2.5Δz_ at different elevations can be derived.

By integrating the extinction coefficient profile on two different elevations z_1_ and z_2_, AOT_Δz_ between two elevations (Δz) can be computed [31] (note: Δz = z_2_ − z_1_):

(1) $${\text{AOT}}_{\Delta \text{z}}=\int_{Z1}^{Z2}\sigma_{\text{a}}(\text{z})\text{dz}$$

The aerosol scaling height z_0_ is defined as the height of an exponential profile at which the value is decreased by 1/e from the ground level value σ_a_(z_0_). It describes the decreasing rate of AOT with altitude and can be calculated using Equation (2) [32,33]:

(2) $${\text{z}}_{0}={\text{AOT}}_{550\text{nm}}/\sigma_{\text{a}}({\text{z}}_{0})$$

where the surface extinction coefficient σ_a_(z_0_) can be derived from the visibility (Equation (3)) [34]:

(3) $$\sigma_{\text{a}}({\text{z}}_{0})=3.912/\text{Vis}(\text{km})$$

Given the surface extinction coefficient σ_a_(z_0_), and insignificances of the aerosol hygroscopic growth effect when relative humidity is less than 70% [35,36], the vertical extinction profile can be estimated:

(4) $$\sigma_{\text{a}}(\text{z})=\sigma_{\text{a}}({\text{z}}_{0})\times \exp(-\text{z}/{\text{z}}_{0})$$

The whole columnar AOT can be divided to AOT_Δz_ by assigning any two given heights (Δz) in Equation (1):

(5) $${\text{AOT}}_{\Delta \text{z}}=\int_{Z1}^{Z2}\sigma_{\text{a}}(\text{z})\text{dz}=\text{AOT}\times [\exp(-{\text{z}}_{1}/({\text{z}}_{0})-\exp(-{\text{z}}_{2}/({\text{z}}_{0}))]$$

In a similar way, visibility at any height Vis_z_ can be calculated from the extinction coefficient by inverting the Koschmeider equation (Equation (3)).

(6) $${\text{Vis}}_{\text{z}}=3.912/\sigma_{\text{a}}(\text{z})$$

Finally, PM_2.5Δz_ concentrations at different elevations can be estimated by applying the linear regression equation (PM_2.5_ = 63.66 × AOT + 26.56) developed by Wong *et al.* [28]:

(7) $${\text{PM}}_{2.5\Delta \text{z}}=63.66\times {\text{AOT}}_{\Delta \text{z}}+26.56$$

**Figure 2.**
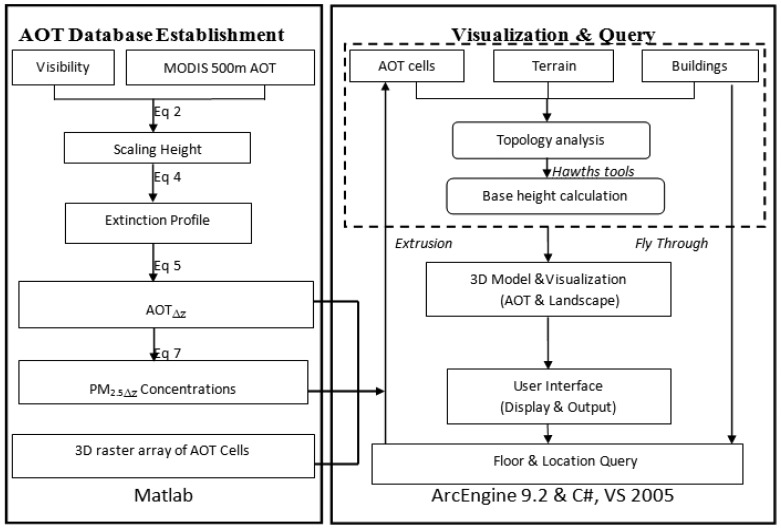
The schematic flow chart of the programs.

A program code in Matlab has been developed for data matching and converting AOT to PM_2.5Δz_. Another program written in ArcEngine helps to display and visualize the data in 3D. The work flow of these programs is shown in Figure 2.

## 4. Results

### 4.1. Correlation between PM_2.5_ Data with Meteorological Data

Table 1 shows the interrelationship between PM_2.5_ and meteorological parameters over Hong Kong on a daily average basis. Moderate correlations were observed between PM_2.5_ and temperature (TEMP), relative humidity (RH), and mean sea level pressure (MSLP), and fair correlations were observed from the other two parameters: wind speed (WS), wind direction (WD).

### 4.2. Diurnal Trend of PM_2.5_ Concentration and Meteorological Data

**Figure 3.**
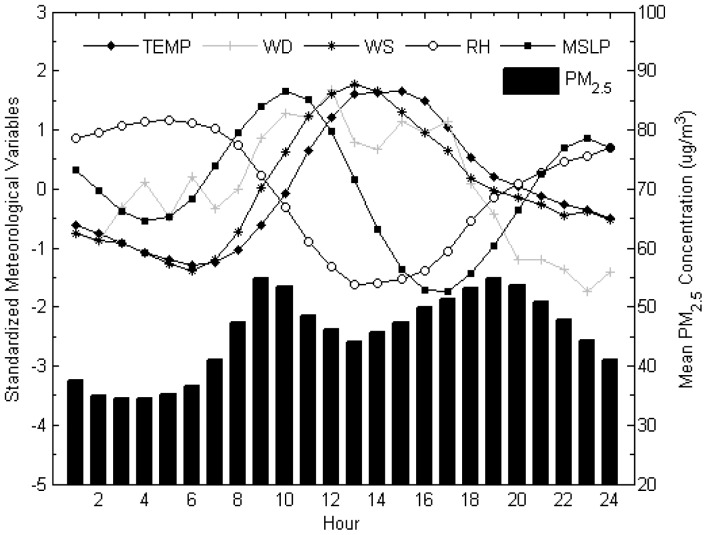
Diurnal trend of PM_2.5_ concentrations and meteorological parameters.

Figure 3 showed the diurnal trends of PM_2.5_ and meteorological parameters. PM_2.5_ showed a distinctive diurnal pattern while low values observed during night time (01:00–05:00). During the daytime, PM_2.5_ exhibited a bimodal pattern with two marked peaks, during morning rush hours (08:00–10:00) and evening rush hours (18:00–20:00), typically when high traffic density occur. Similar observations and implications were reported by Chan and Kwok [37].

Wind direction does not show a clear diurnal pattern. Mean sea level pressure has a similar pattern to that of PM_2.5_ in spite of the time lag, which displayed two clear maxima around 10:00 and 23:00. In contrast, temperature and wind speed exhibit a unimodal pattern characterized by midday maxima around 13:00. Relative humidity, however, exhibits an inverse unimodal pattern with stable overnight maximum values, which suggests the negative association with nocturnal PM_2.5_ concentrations. A high relative humidity can depress the absorption of gas phase organic species into particle surface [38] and accelerate the removal of particle by dry deposition, this mechanism enhanced for hygroscopic particle [39]. Thus, PM_2.5_ keeps constant at minimum values between 02:00 and 05:00. Another reason is due to less influence of anthropogenic activities on fine particulate levels during nighttime.

**Figure 4.**
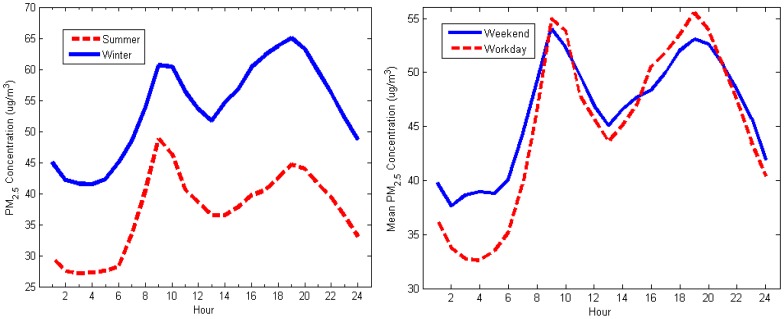
Diurnal trends of PM_2.5_ concentrations (**left**) during summer and winter; and (**right**) during weekend and weekday.

Despite a similar pattern observed in Figure 4 (left), summer diurnal PM_2.5_ concentrations is found to be lower than in winter. On the other hand, the peak values in Figure 4 (right) are higher on weekdays compared with weekends, which may be caused by more anthropogenic activities.

### 4.3. Monthly and Seasonal Trends of PM_2.5_ Concentration and Meteorological Data

**Figure 5.**
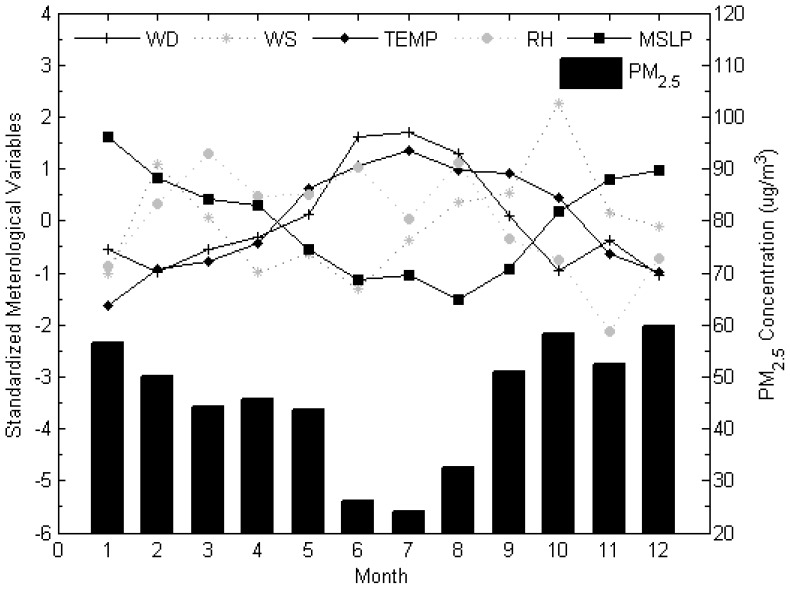
Seasonal variations of PM_2.5_ concentrations and meteorological parameters.

Seasonal variations of PM_2.5_ were obvious (Figure 5). The concentrations are higher in winter and autumn and lower in spring and summer seasons. Previous studies of roadside suspended particulates at heavily trafficked urban areas in Hong Kong conducted in 2000 [37] and 2005 [40] showed similar seasonal patterns. The mean sea level pressure exhibits a similar pattern as PM_2.5_ characterized.

### 4.4. 3D PM_2.5_ Visualization and Query Prototype

**Figure 6.**
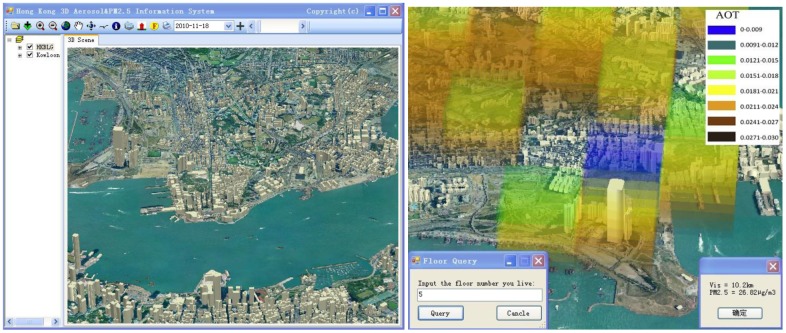
Screenshot of (**left**) user interface, visualizing Hong Kong with the extruded building in 3D; and (**right**) example of PM_2.5_ query (adopted from [30]).

In order to understand the spatial pattern of PM_2.5_ concentrations in the context of the built-up environment of Hong Kong, a Geographic Information Systems (GIS) prototype was developed in this study to integrate atmospheric PM_2.5_ vertical profiles with 3D GIS data which are provided by the Hong Kong Lands Department. This prototype utilized ESRI ArcGIS Scene Control component to present the landscape objects including the terrain model, building polygons, AOT_Δz_ and PM_2.5Δz_ grid data in 3D space. The functionality of this system provides scene rendering using perspective view. The 3D PM_2.5Δz_ atmospheric layers corresponding to 500 m pixel columns were rendered using a transparent color scheme overlaid with the 3D building polygons. Since the system is aimed at the built environment within the city, only seven PM_2.5Δz_ atmospheric layers, each has 75 m elevation, were created. In this GIS prototype, each building is corresponding with its cadastral footprint polygon, which owns attributes including building height, number of floors and height of each floor (e.g., building height/number of floors). The PM_2.5Δz_ data can be related with each building by tabular linkage through the polygon-in-polygon function of the Hawths extension [41]. Therefore, any floor of a building can be related to the PM_2.5Δz_ concentrations and useful for direct query. The interface of this GIS prototype is shown in Figure 6 (left). Figure 6 (right) shows the query results of the PM_2.5Δz_ concentrations of the International Commerce Centre on 1 February 2007 (local time 10:50).

## 5. Discussion and Conclusions

The paper presents a comprehensive study of characteristics, behavior and trends of PM_2.5_, as well as its correlation with different meteorological parameters and the state-of-the-art technique for modeling and visualizing of atmospheric PM_2.5Δz_ vertical profiles. In this study, the hourly based dataset, e.g., PM_2.5_ concentrations and five meteorological parameters e.g., wind direction, wind speed, temperature, relative humidity, and pressure were analyzed to explore their diurnal and seasonal variations and interrelations.

PM_2.5_ showed a distinctive bimodal pattern with two marked peaks: morning rush hours (08:00–10:00) and evening rush hours (18:00–20:00), which are mostly influenced by the dense traffic. The lower PM_2.5_ concentrations observed in summer than in winter may be caused by the wind direction. Northerly and northeasterly winds bring pollutants from the Chinese mainland in winter, whereas southerly monsoon winds from the sea bring fresh air to the city in summer. In addition, the higher concentrations of PM_2.5_ in rush hours on weekdays compared to those in weekends suggest the significance of anthropogenic activities e.g., traffic-related local PM_2.5_ emissions.

The PM_2.5Δz_ values for different atmospheric heights were linked to a GIS-based 3D urban model to provide near-real time visualization. The resulting 3D database of PM_2.5Δz_ concentrations provides crucial information to air quality regulators and decision makers to comply with air quality standards and in devising control strategies. This prototype will be integrated with web-interface system in the near future.

## Figures and Tables

**Table 1.** Correlation coefficient of PM_2.5_ and meteorological factors for 2007 and 2008.

**Correlation (r)**	**PM_2.5_**	**WD**	**WS**	**TEMP**	**RH**	**MSLP**
**PM_2.5_**	1.000	−0.101	0.095	−0.478	−0.366	0.504
**WD**	−0.101	1.000	−0.683	0.291	−0.052	−0.342
**WS**	0.095	−0.683	1.000	−0.220	0.011	0.222
**TEMP**	−0.478	0.291	−0.220	1.000	0.083	−0.866
**RH**	−0.366	−0.052	0.011	0.083	1.000	−0.338
**MSLP**	0.504	−0.342	0.222	−0.866	−0.338	1.000
